# Metabolomic Analysis of Severe Osteoarthritis in a Spanish Population of Women Compared to Healthy and Osteoporotic Subjects

**DOI:** 10.3390/metabo12080677

**Published:** 2022-07-24

**Authors:** Clara Pertusa, Damián Mifsut, José Manuel Morales, Juan J. Tarín, Antonio Cano, Daniel Monleón, Miguel Ángel García-Pérez

**Affiliations:** 1 Research Unit, INCLIVA Health Research Institute, 46010 Valencia, Spain; clara.pertusa@uv.es (C.P.); mifsut.dam@gmail.com (D.M.); j.manuel.morales@uv.es (J.M.M.); 2 Orthopedic Surgery and Traumatology, Clinic Hospital, INCLIVA Institute of Health Research, 46010 Valencia, Spain; 3 Department of Pathology, University of Valencia, 46010 Valencia, Spain; 4 Department of Cellular Biology, Functional Biology and Physical Anthropology, University of Valencia, 46100 Burjassot, Spain; juan.j.tarin@uv.es; 5 Department of Pediatrics, Obstetrics and Gynecology, University of Valencia, 46010 Valencia, Spain; antonio.cano@uv.es; 6 Center for Biomedical Research Network on Frailty and Health Aging (CIBERFES), 28029 Madrid, Spain; 7 Department of Genetics, University of Valencia, 46100 Burjassot, Spain

**Keywords:** osteoarthritis, osteoporotic fracture, metabolomic analysis, postmenopausal women

## Abstract

Bone pathologies such as osteoporosis (OTP) and osteoarthritis (OA) are rising in incidence with the worldwide rise in life expectancy. The diagnosis is usually obtained using imaging techniques such as densitometry, but with both being multifactorial diseases, several molecular mechanisms remain to be understood. Metabolomics offers the potential to detect global changes which can lead to the identification of biomarkers and a better insight in the progress of the diseases. Our aim was to compare the metabolic profiles of a cohort of 100 postmenopausal women, including subcapital hip fragility fracture patients, women with severe OA of the hip that required the implantation of a hip prosthesis and controls, to find altered metabolites and networks. Nuclear magnetic resonance (NMR) spectroscopy was used to obtain the metabolomic profiles of peripheral blood derived serum, and statistical analysis was performed using MATLAB V.6.5. 30 of the 73 metabolites analysed showed statistically significant differences in a 3-way ANOVA, and 11 of them were present in the comparison between OA and controls after adjustment by covariates, including amino acids, energy metabolism metabolites and phospholipid precursors. PLS-DA analysis shows a good discrimination between controls and fracture subjects with OA patients, and ROC curve analysis demonstrates that control and fracture subjects were accurately discriminated using the metabolome, but not OA. These results point to OA as an intermediate metabolic state between controls and fracture, and suggest that some metabolic shifts that happen after a fracture are also present at weaker intensity in the OA process.

## 1. Introduction

Osteoarthritis (OA) and osteoporosis (OTP) are two of the most prevalent musculoskeletal diseases in the elderly in western populations, both causing serious medical and socioeconomic consequences. These diseases, whose sequelae range from pain and deformity to fractures, dramatically affect patient functional status, and with the increase in life expectancy, they are predicted to become predominant causes of disability worldwide [1].

OA and OTP are multifactorial pathologies and their onset is influenced by genetic and environmental factors, and their interactions [2]. Both share common risk factors, such as age, sex, or inflammatory states. However, there are certain risk factors which differ between the two of them, such as bone mass index (BMI), bone mineral density (BMD) and mechanical load [3].

The main causes for the development of OTP are aging and the estrogenic depletion that occurs during menopause [4]. These processes cause the loss of bone mass and the deterioration of the microarchitecture of bone tissue, which causes decay in bone quality [4]. This leads to an increase in the risk of fragility fractures, which is a factor of the first magnitude in morbidity and mortality [5]. Effectively, at age 50, the percentage of women with OTP in the European Union (EU) who will suffer a fracture of the hip, spine, forearm, or proximal humerus in the next 10 years is approximately 45% [6]. Importantly, among the main types of fracture, hip fracture is undoubtedly the most disabling and with worse prognosis fracture because it is associated with high rates of morbidity and mortality [7]. 

The hallmark of OA is the alteration of cartilage, though this concept has changed, and OA is now considered a disease of the whole joint. Effectively, OA is a progressive disorder characterized by cartilage degradation, osteophyte formation, joint space narrowing, and subchondral sclerosis [8]. However, OA is a heterogeneous disease with respect to age of onset, number of affected joints, systemic or local risk factors, bone implication and inflammation. In fact, with respect to bone implication, three types of OA can be described: osteoporotic, bone-forming, and erosive [3]. The risk factors for OA include age, female sex, adiposity, joint biomechanics, repetitive joint use, and genetic factors, with the prevalence in north American and European adults above 65 years old being 60%, 33% and 5% for OA hands, knee and hip, respectively [8].

There is currently great interest in identifying biomarkers that can inform about the pathogenesis of an illness. A biomarker represents a quantifiable characteristic of a normal or pathologic biological process and can help identify the early stages, progress, and response to treatment in a disease [9]. The most common biomarkers studied to this date include cytokines, miRNAs and, of course, metabolites [10]. 

Metabolomics, which represents the global profiling of metabolites in biological fluids, cells, and tissues, has the potential to discover new biomarkers for diseases [11]. Metabolomics can be used to capture the global changes in networks and altered biochemical pathways due to a pathological process, even in early stages of the disease, which can lead to the identification of biomarkers for the process. Nuclear magnetic resonance (NMR) spectroscopy is one of the technological options to analyse the metabolic profile. In recent years, various studies have performed a metabolomic approach using NMR to identify discriminatory metabolic profiles for a vast number of diseases, including OTP [12] and OA [13]. 

In the present study we have used NMR to analyse serum metabolites in women with severe hip OA and fragility hip fracture, both requiring prosthetic hip placement, and control postmenopausal women. Our aim in comparing the metabolic profiles of women with and without OA with those of fracture patients was the identification of common and distinctive metabolic profiles which can inform about metabolic networks altered in these conditions. 

## 2. Results

### 2.1. General Characteristics of the Population

The study participants’ anthropometric and bone characteristics are shown in Table 1.

Women in the OA and Frac groups were significantly older than those in the Ctrl group, and women in the Frac group were also significantly older than women in the OA group. The three groups did not show significant differences in BMI (*p* = 0.957). As can be expected, there were major differences between groups in femoral neck bone parameters, with women in the Frac group showing significantly worse bone parameters than those in Ctrl and OA groups. Although not significant, the OA group shows a clear trend towards higher BMD with respect to the Ctrl group, with *p* = 0.095 for FN-BMD, *p* = 0.053 for FN T-score and *p* = 0.1 for FN Z-score. No significant differences between groups were found for LS-BMD related parameters.

Table 2 displays the biochemical characteristics of the cohort. There were statistically significant differences between groups for cholesterol, HDL and LDL, with OA and Frac showing lower values than Ctrl in all three parameters. Regarding bone metabolism parameters, all of them showed significant differences between groups, with Total ALP and 25(OH)D3 being decreased in OA and Frac with respect to Ctrl; and CTx being elevated in OA and Frac with respect to control. CTx is the only biochemical marker in which we were able to find significant differences between OA and Frac, with the Frac group presenting higher levels.

### 2.2. Metabolic Profiles

We quantified 73 metabolic spectral features in blood plasma samples from 53 control subjects, 23 osteoarthritic subjects and 27 subjects after fracture. Thirty of these metabolites show statistically significant differences in a three-way ANOVA (Table 3).

**Figure 1:**
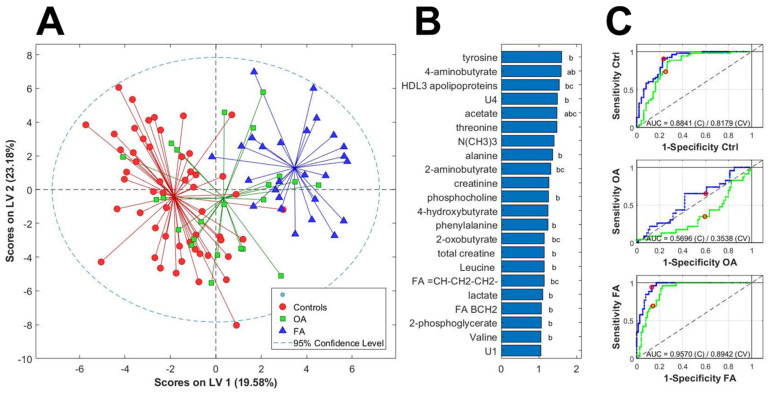
(**A**) Scores plot of the PLS-DA analysis of the metabolome, adjusted by age, BMI and BMD for the discrimination between Ctrl group (red circles), OA (green squares) and Frac (blue triangles). (**B**) Metabolites with PLS-DA VIP score higher than 1 for the same PLS-DA model of the metabolome. a: OA vs. Ctrl group; b: Frac vs. Ctrl group; c: Frac vs. OA group. (**C**) Receiver-operating curve (ROC) analysis for the classification of each individual group (from top to bottom; Ctrl group, OA group and Frac group based on our PLS-DA model.

Many of these differences were not present when the data was adjusted for age. BMI and BMD suggesting, as previously reported, an important influence of these factors in the plasma metabolome. Only 11 of these significant differences were present in the adjusted comparison between OA and controls. Differences included amino acids; energy metabolism metabolites; some bacterial co-metabolism metabolites such as methylamines, acetate and butyrate derivatives; and phospholipids precursors. Tyrosine appears as the metabolite with strongest associations both in OA group and Frac group. The scores plot of the PLS-DA analysis (Figure 1A) of all the samples in this model show a good discrimination between controls and fracture subjects with OA patients in a transition region overlapping with both groups. Twenty out of the 30 significant metabolites also show a PLS-DA VIP score higher than 1 (Figure 1B). The Receiver-operating curve analysis for the classification of each individual group based on our metabolome PLS-DA model (Figure 1C) shows that control (AUC = 0.88) and fracture subjects (AUC = 0.96) were accurately discriminated using the metabolome both in training and cross-validation. However, the model did not show any value in discriminating OA patients in this context (AUC = 0.57) due to the high overlapping with the other two groups.

## 3. Discussion

The identification and management of OA remains a clinical challenge. Diagnosis is usually achieved by imaging techniques after the apparition of symptoms such as pain and stiffness; and current treatments, whether they are oral or intraarticular, aim to reduce these symptoms and preserve joint function [14]. However, correlation between the symptoms and the pathological severity of OA is low, so better mechanistic knowledge is currently needed [15]. Our study aimed to explore the metabolomic profiles in women with and without OA and compare them with fracture patients to identify common patterns and better risk biomarkers for these conditions, mainly in OA. In this work we have studied a cohort of postmenopausal women including healthy subcapital hip fragility fracture patients and women with severe OA of the hip that required the implantation of a hip prosthesis, without other comorbidities. Serum samples from all of them were analysed with NMR to obtain the metabolic profile. To analyse discrimination among groups, PLS-DA and ROC curve analysis were performed, and MSEA analysis was performed to identify significant changes in metabolic networks. 

Although previous studies have explored metabolomic profiles of OA [16], this is the first time that multivariate PLS-DA models adjusted for confounders, namely age, BMI and BMD have been used to detect associations and reduce false positive discoveries. Our multivariate PLS-DA models adjusted for age, BMI and BMD revealed that although differentiation between OA and controls seems difficult and is not possible with just metabolomics, OA represents a middle state between controls and fracture. Although the interpretation of these results is far from simple, the scores plot suggests that some of the metabolic shifts that happen after a fracture are also present at weaker intensity in the OA process. Moreover, twenty metabolites show statistical association with OA and/or fracture and had high contribution (VIP greater than 1) to the discrimination models. 

**Figure 2:**
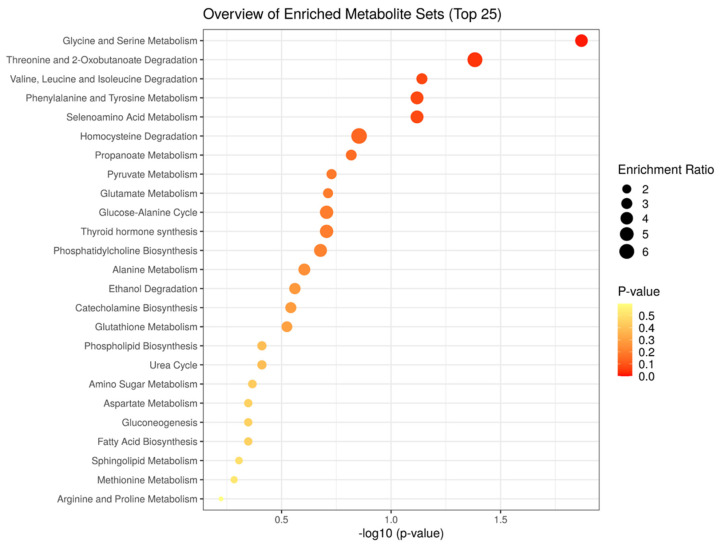
The metabolite set enrichment analysis of our metabolites with PLS-DA scores higher than 1 and *p*-values < 0.05 after the analysis with MetaboAnalyst. Metabolic pathways whose name is indicated are significant (*p*-value lower than 0.05 after the adjustment using Holm-Bonferroni method and False Discovery Rate) and have a pathway impact value calculated from pathway topology analysis over 0. The pathways are represented as circles. The circle color indicates the significance level from highest (red) to lowest (white) in the enrichment analysis. The circle size is proportional to the impact value of each road from the topology analysis.

The metabolites identified as relevant in this study cover a wide spectrum of compounds in the organism. In general, it is well-known that metabolic homeostasis and regulation worsens with age [17]. Loss of muscle to fat mass ratio contributes to this dysregulation and explains some of the metabolic consequences of age [18]. We observed that OA further decreases total creatine in blood, typically associated to muscle catabolism. Creatine plays an important role in muscle contraction by transferring of phosphoryl groups to regenerate ATP through a reversible reaction catalyzed by phosphocreatine kinase. The increase in blood lactate observed in OA in our study further supports a loss of muscle metabolic function in our patients. Glycine and serine metabolism is the most significant metabolic core associated to these three groups in our metabolite set enrichment analysis. Glycine is a major component of collagen, which in turn is a major component of cartilage and joints. Tyrosine and phenylalanine are among the metabolites associated to OA and with high contribution to the models. These aromatic metabolites are major precursors of some catecholamines, and their changes may be related to stressful situations. In addition, phenylalanine has been reported in many studies as a marker of metabolic health [19]. The metabolite set enrichment analysis of our metabolites with PLS-DA scores higher than 1 and *p*-values < 0.05 (Figure 2) also show a predominant role of phenylalanine and tyrosine metabolism in the impact of OA. Some studies demonstrated local production of catecholamines in OA patients for reducing inflammation [20]. Our findings suggest that this local synthesis mobilize catecholamines precursors in blood. Overall, these results represent novel metabolomic findings associated to well-known pathophysiological events in the onset and progression of OA.

Leucine and valine are branched chain amino acids (BCAA) typically associated with metabolic diseases e.g., type 2 diabetes or obesity [21], and are altered in our OA patients both at individual level and at metabolite set enrichment level. BCAA have already been suggested as potential biomarkers for OA [22]. Although people who have type 2 diabetes have an increased risk of OA, this association has been traditionally attributed to underlying shared risk factors such as age and obesity (mainly to obesity—a risk factor for type 2 diabetes) rather than to the diabetes itself. However, recent studies suggest that not only the increased inflammation state associated with diabetes mellitus can escalate the breakdown of joint tissues; but also lipid metabolism and glycemia alterations directly impact on cartilage health and subchondral bone [23] accelerating the progression of the disease and worsening the reported pain. The accumulation of advanced glycation end products seems to have an effect on the mechanical structure of tendons, inhibiting the differentiation and promoting the apoptosis of tendon-derived stem cells and interfering with the type I collagen organization in the extracellular matrix [24]. Our findings on leucine and valine combined with the results in other metabolites related to glucose metabolism, even when adjusted for BMD, BMI, and age, further support a potential role of type 2 diabetes on OA beyond obesity and age. The differences observed in total lipids and cholesterol in the OA group also back a role of metabolic disease in the development of OA and open new hypotheses to explore in the management and early detection of bone and cartilage related health problems. 

The gut microbe ecosystem modulates and shapes many metabolic, immunological, structural, and neurological functions [25]. Alterations in gut microbiota and the host-microbiota co-metabolism are involved in the initiation and progression of inflammation-driven diseases [26]. Our results show alterations and relevant roles of many of these host-microbiota co-metabolism in the context of OA. Butyrates derivatives e.g., 2- and 4- aminobutyrates and 2-oxobutyrate are co-metabolites that are initially produced in the gut in the form of short chain fatty acids (acetate, Propionate, and butyrate) and are further processed by human cells. Phosphocholine is also a metabolite that partially comes from the processing in the gut of dietary carnitine into trimethylamines and cholines. The metabolite set enrichment analysis also shows threonine and 2-oxobutyrate degradation, closely related to bacterial co-metabolism, as the second most significantly affected metabolic core (Figure 2). The identification of changes in these co-metabolites in OA suggests an important role of alterations in gut microbiota and its pro-inflammatory effects in the onset and progression of OA.

This study presents some limitations. All the cohorts are comprised of Caucasian women, so the data obtained may not be fully extrapolated to men or other populations. The difficulty of obtaining subjects has caused the sample size to be modest and the groups to have different number of subjects, and we lack data on other co-variables such as the dietary habits, diabetic status, or physical activity of the participating women. Also, there are significant age differences among groups, which is why it was selected as a covariate for the adjustment. Finally, with this being a case-control study, all the results presented here are statistical associations and further study will be necessary to determine whether these associations are causal. 

As a conclusion, in this article we present an analysis of the main altered circulating metabolites in healthy, OTP and OA patients, which point to OA as an intermediate state between control and fracture; an interpretation that opens paths for further study. Our findings also back up the current studies linking type 2 diabetes as a trigger for OA, proving the utility of metabolomics in the investigation of the underlying etiology of the disease.

## 4. Experimental Design

### 4.1. Study Subjects

All participating subjects were postmenopausal Caucasian women living in Valencia, Spain, recruited from the Hospital Clínico Universitario. The patients were consecutively enrolled in the cohort as Fracture Group (Frac Group), OA Group and Control Group (Ctrl Group).

The subjects included in the Frac group were 27 women with subcapital hip fragility fracture. 23 women with severe OA of the hip that required the implantation of a hip prosthesis were included in the OA group. The Ctrl group was comprised of 53 women without bone pathologies. Exclusion criteria in this study were: (i) having a history of bone disease other than primary OTP or OA. (ii) hip fracture due to high-energy trauma (iii) chemotherapy before densitometric study (iv) previous use of any medication known to alter bone metabolism, such as corticosteroid treatment, and (v) being under 50 years of age. 

The study was approved by Clinical Research Ethics Committee of our institution, and the participants read and signed an informed consent according to the guidelines of the Institute of Health Research; INCLIVA. 

### 4.2. Anthropometric, Biochemical and Bone Density Measurements

Blood samples under fasting conditions were collected from all participants to obtain serum, which was stored at −80 °C until used. 

The levels of carboxy-terminal telopeptides of type I collagen (CTx) and 25-Hydroxycholecalciferol (25(OH)D3) were measured by electrochemiluminescence (E170 Modular Analyzer, Roche Diagnostics, Mannheim. Germany). Levels of total alkaline phosphatase (ALP), glucose, HDL, LDL, cholesterol, and triglycerides were determined by routine methods using an autoanalyzer (Olympus 5400. Tokyo. Japan).

Bone mineral density (BMD) quantification was performed in both the lumbar spine (L2-L4, LS-BMD) and at the non-dominant proximal end of the femoral neck (FN-BMD), except if this were the site of fracture, using dual energy X-ray absorptiometry (DXA) with a Lunar DPX densitometer (GE Lunar Corporation. Madison, WI, USA), a Norland XR-36 (Norland Medical Systems Inc; Fort Atkinson, WI, USA), or Hologic (Hologic Explorer. Marlborough, MA, USA) densitometers. Standardized BMD (sBMD) was calculated for comparison between subjects [27].

The BMI was calculated for each participant as weight (kg) divided by height squared (m^2^).

### 4.3. NMR Metabolomics

A single pulse presaturation experiment was acquired in all samples. The number of transients was 256 collected into 65 k data points for all experiments. Spectral chemical shift referencing on the alanine CH3 doublet signal at 1.475 ppm was performed in all spectra. Spectra were processed using MestReNova 8.1 (Mestrelab Research S.L., Santiago de Compostela, Spain) and transferred to MATLAB (MathWorks. 2012) using in-house scripts for data analysis. 

The chemical shift region including resonances 0.50–4.70 ppm (the aliphatic region) and 5.20–10.00 ppm (the aromatic region) was investigated. Metabolite spin systems and resonances were identified by literature data and Chenomx resonances database (Chenomx NMR 7.6). Spectra were normalized to total aliphatic spectral area, with lipid excluded to eliminate differences in metabolite total concentration. NMR peaks were integrated and quantified using semi-automated in-house MATLAB peak-fitting routines. Final metabolite relative spectral abundance was calculated in arbitrary units as peak area normalized to total spectral area. Chemometrics analysis were performed with PLS ToolBox 8.0 (Eigenvector Inc., Wenatchee, WA, USA) in MATLAB. Finally, each metabolic feature was normalized to the standard deviation in all the samples to obtain z-scores. 

To maximize the separation between samples and to identify discriminant patterns, partial least-squares discriminant analysis (PLS-DA) was applied. We adjusted the analysis for age, BMI and BMD by calculating a linear regression model with these 3 variables for each metabolic feature and using the residues for the PLS-DA analysis. A permutation test was performed to check overfitting of the PLS-DA models. The multivariate chemometric models were cross-validated with 10-fold Venetian blind cross-validation. In each run, 10% of data were left out of the training and used to test the model. Spectral regions with high variable importance in projections (VIP) coefficients obtained during PLS-DA are more important in providing class separation during analysis, while those with very small VIP coefficients provide little contribution to classification. A Metabolite Set Enrichment Analysis (MSEA) over metabolites with VIPs scores higher than 1 and *p*-values below 0.05 was performed with MetaboAnalyst, and both the Small Molecule Pathway Database (SMPDB) and the blood samples disease database. MSEA is conceptually similar to Gene Set Enrichment Analysis and uses a collection of predefined metabolites sets to rank the lists of metabolites obtained from metabolomics studies. By using this prior knowledge about metabolite sets, we could identify significant and coordinated changes in metabolic networks and obtain biological insight.

### 4.4. Statistical Analysis

Fixed-effects analysis of variance (ANOVA) designs was used to compare means between groups. Analysis of covariance (ANCOVA) was used to examine differences in the dependent variables (metabolites) among groups after adjustment for confounding variables. Age, BMI and BMD were considered as covariates. Levene’s test was used to assess the homogeneity of variances for each dependent variable across all level combinations of the between-subject factors. Bonferroni’s test (when the variances were assumed to be equal) and Dunnett’s T3-test (when the variances were assumed to be unequal) were applied to perform post-hoc pairwise multiple comparisons between groups. Data was analysed using IBM SPSS statistics for Windows (v.26.0; IBM Corp., Armonk, NY, USA).

## Figures and Tables

**Table 1:** Anthropometric and bone characteristics of the women in the study cohort.

Subject Characteristics	Control (N = 53)	Osteoarthritis (N = 23)	Fracture (N = 27)	ANOVA *p*-Value
Age (years)	70.02 ± 7.13	76.00 ± 9.49 (a)	83.22 ± 7.73 (b, c)	<0.000
Weight (Kg)	69.50 ± 11.51	69.50 ± 15.13	68.62 ± 11.77	0.955
Height (cm)	157.67 ± 6.47	158.35 ± 6.25	157.00 ± 4.92	0.795
BMI (kg/m^2^)	28.04 ± 4.83	27.66 ± 5.53	27.81 ± 4.42	0.957
FN-BMD (g/cm^2^)	0.79 ± 0.16	0.87 ± 0.12	0.62 ± 0.11 (b, d)	<0.000
FN T-score	−1.10 ± 1.43	−0.22 ± 1.11	−2.50 ± 0.97 (b, d)	<0.000
FN Z-score	0.23 ± 1.43	0.99 ± 1.15	−0.80 ± 0.71 (b, d)	<0.000
LS-BMD (g/cm^2^)	1.01 ± 0.16	1.05 ± 0.18	0.95 ± 0.20	0.226
LS T-score	−1.15 ± 1.44	−0.78 ± 1.56	−1.66 ± 1.70	0.240
LS Z-score	0.45 ± 1.58	0.75 ± 1.59	0.41 ± 1.70	0.803

BMI: body mass index, BMD: bone mineral density, FN: femoral neck, LS: lumbar spine. (a): *p* < 0.01 vs. control, (b): *p* < 0.000 vs. control, (c): *p* < 0.01 vs. osteoarthritis, (d): *p* < 0.000 vs. osteoarthritis.

**Table 2:** Biochemical characteristics of the study cohort.

Metabolites	Control (N = 53)	Osteoarthritis (N = 23)	Fracture (N = 27)	ANOVA *p*-Value
CTx (ng/mL)	0.347 ± 0.147	0.457 ± 0.156 (a)	0.709 ± 0.306 (b, c)	<0.000
Total ALP (U/L)	88.4 ± 32.3	145.0 ± 81.1 (a)	176.8 ± 96.4 (b)	<0.000
25(OH)D3 (ng/mL)	35.3 ± 52.4	15.2 ± 9.1	14.8 ± 14.0	0.043
Cholesterol (mg/dL)	205.4 ± 32.8	158.4 ± 38.8 (b)	141.9 ± 36.1 (b)	<0.000
Triglycerides (mg/dL)	105.1 ± 43.5	113.8 ± 43.3	115.2 ± 48.9	0.585
HDL (mg/dL)	62.8 ± 14.6	47.3 ± 13.9 (b)	39.6 ± 9.1 (b)	<0.000
LDL (mg/dL)	124.8 ± 30.5	97.0 ± 25.1 (a)	88.8 ± 27.8 (b)	<0.000
Glucose (mg/dL)	112.4 ± 31.4	109.3 ± 27.6	127.4 ± 30.4	0.100

CTx: carboxy-terminal telopeptides of collagen I, ALP: alkaline phosphatase, 25(OH)D3: 25-hydroxycholecalciferol. (a): *p* < 0.05 vs. control, (b): *p* < 0.000 vs. control, (c): *p* < 0.01 vs. osteoarthritis.

**Table 3:** Mean value and standard deviation (SD) for metabolic relative abundance spectral features z-scores and *p*-values for non-adjusted (No Adj.) and age, BMI and BMD adjusted (Adj.) comparison between controls, osteoarthritic patients (OA) and fracture patients (FA).

-	Metabolites-	Control	OA	FA	3 Way		Control vs. OA		FA vs. OA		Control vs. FA	
		(N = 53)	(N = 23)	(N = 27)	*p*-Value		*p*-Value		*p*-Value		*p*-Value	
Amino acids		**Mean ± SD**	**Mean ± SD**	**Mean ± SD**	**No Adj.**	**Adj.**	**No Adj.**	**Adj.**	**No Adj.**	**Adj**	**No Adj.**	**Adj.**
	Isoleucine	0.278 ± 0.90	−0.032 ± 0.91	−0.518 ± 1.08	0.003	0.087	0.174	0.164	0.096	0.639	0.001	0.105
	Alanine	0.367 ± 0.86	−0.009 ± 0.95	−0.713 ± 0.92	0.000	0.000	0.096	0.020	0.011	0.743	0.000	0.000
	Leucine	0.295 ± 0.93	0.039 ± 0.98	−0.613 ± 0.90	0.000	0.001	0.280	0.062	0.018	0.594	0.000	0.000
	Glutamate	0.172 ± 1.05	0.101 ± 0.93	−0.423 ± 0.86	0.035	0.045	0.783	0.443	0.043	0.716	0.013	0.012
	Glutamine	0.195 ± 1.03	0.080 ± 0.99	−0.450 ± 0.82	0.021	0.018	0.652	0.306	0.044	0.782	0.006	0.005
	Aspartate	0.126 ± 1.02	0.027 ± 0.98	−0.270 ± 0.96	0.246	0.360	0.698	0.419	0.284	0.289	0.099	0.129
	Glycine	0.241 ± 0.86	−0.179 ± 1.23	−0.320 ± 0.94	0.036	0.049	0.093	0.047	0.650	0.403	0.009	0.073
	Threonine	0.387 ± 0.83	0.003 ± 1.08	−0.761 ± 0.80	0.000	0.000	0.097	0.057	0.006	0.838	0.000	0.000
	Valine	0.302 ± 0.87	−0.064 ± 0.94	−0.539 ± 1.08	0.001	0.042	0.104	0.081	0.107	0.648	0.000	0.067
	Total creatine	−0.319 ± 0.80	0.124 ± 1.23	0.522 ± 0.92	0.001	0.026	0.066	0.054	0.198	0.543	0.000	0.023
	Phenylalanine	0.266 ± 0.68	0.229 ± 1.50	−0.717 ± 0.60	0.000	0.000	0.883	0.015	0.004	0.694	0.000	0.000
	Tyrosine	0.433 ± 0.85	−0.043 ± 0.97	−0.813 ± 0.78	0.000	0.000	0.035	0.030	0.003	0.807	0.000	0.000
Cholesterol and lipoproteins	Cholesterol	0.208 ± 1.09	−0.034 ± 0.90	−0.380 ± 0.80	0.043	0.549	0.352	0.507	0.156	0.981	0.015	0.557
	HDL apolipopr	0.410 ± 0.87	−0.023 ± 0.99	−0.786 ± 0.77	0.000	0.001	0.060	0.057	0.004	0.780	0.000	0.001
Fatty acids	FA -CH_3_	0.255 ± 1.04	−0.043 ± 0.86	−0.464 ± 0.87	0.008	0.336	0.234	0.417	0.093	0.877	0.003	0.360
	FA BCH_2_	0.291 ± 0.90	−0.026 ± 0.97	−0.548 ± 1.01	0.001	0.014	0.173	0.082	0.070	0.672	0.000	0.012
	FA =CH-CH_2_-CH_2_-	0.341 ± 0.93	−0.126 ± 0.94	−0.562 ± 0.93	0.000	0.022	0.048	0.066	0.108	0.597	0.000	0.038
	FA a-CH_2_	0.183 ± 0.96	−0.061 ± 0.98	−0.308 ± 1.04	0.108	0.142	0.316	0.189	0.394	0.504	0.039	0.087
	FA-CH = CH	0.180 ± 0.98	−0.159 ± 1.02	−0.218 ± 0.99	0.168	0.735	0.176	0.421	0.836	0.153	0.092	0.890
	Valerate	0.218 ± 0.99	−0.035 ± 0.99	−0.399 ± 0.94	0.031	0.031	0.308	0.149	0.189	0.804	0.009	0.015
Energy metabolism—glycolisis	Glucose	0.188 ± 0.88	0.198 ± 1.32	−0.537 ± 0.70	0.004	0.128	0.969	0.515	0.015	0.861	0.000	0.107
	Lactate	0.314 ± 0.81	−0.082 ± 1.10	−0.546 ± 1.04	0.001	0.022	0.084	0.043	0.132	0.483	0.000	0.019
	2-phosphoglycerate	−0.224 ± 0.61	−0.222 ± 1.26	0.628 ± 1.14	0.000	0.015	0.995	0.339	0.015	0.414	0.000	0.001
Energy metabolism—ketone bodies	3-hydroxybutyrate	0.253 ± 1.07	−0.109 ± 0.84	−0.403 ± 0.85	0.016	0.430	0.155	0.289	0.226	0.638	0.007	0.623
	Acetate	0.409 ± 0.86	−0.050 ± 0.95	−0.760 ± 0.87	0.000	0.000	0.041	0.021	0.008	0.924	0.000	0.000
Fluid balance	Creatinine	0.336 ± 0.88	−0.007 ± 1.09	−0.654 ± 0.84	0.000	0.004	0.151	0.105	0.022	0.701	0.000	0.002
Inflammation	Glycoprotein A	0.091 ± 0.86	−0.072 ± 1.06	−0.117 ± 1.21	0.634	0.783	0.480	0.458	0.892	0.196	0.378	0.975
Bacterial co-metabolism	4-hydroxybutyrate	0.325 ± 0.92	0.020 ± 0.95	−0.655 ± 0.90	0.000	0.001	0.192	0.064	0.013	0.690	0.000	0.000
	2-aminobutyrate	0.354 ± 0.89	−0.009 ± 0.97	−0.687 ± 0.90	0.000	0.000	0.116	0.031	0.014	0.980	0.000	0.000
	4-aminobutyrate	0.406 ± 0.74	0.070 ± 1.05	−0.856 ± 0.89	0.000	0.000	0.116	0.008	0.001	0.494	0.000	0.000
	2-oxobutyrate	0.313 ± 0.93	−0.040 ± 0.92	−0.580 ± 0.97	0.001	0.071	0.131	0.148	0.050	0.751	0.000	0.105
	N(CH_3_)_3_	0.375 ± 0.93	−0.089 ± 1.00	−0.659 ± 0.78	0.000	0.001	0.054	0.015	0.029	0.717	0.000	0.010
	Dimethylamine	0.056 ± 0.35	0.345 ± 1.97	−0.404 ± 0.32	0.024	0.000	0.302	0.049	0.058	0.622	0.000	0.000
Phospholipids precursors	Phosphocholine	0.275 ± 0.52	0.223 ± 1.63	−0.730 ± 0.61	0.000	0.000	0.834	0.002	0.007	0.572	0.000	0.000
	Choline	0.107 ± 0.76	−0.029 ± 1.26	−0.186 ± 1.17	0.462	0.634	0.562	0.733	0.650	0.895	0.179	0.265
Unknowns	U1	0.212 ± 0.93	0.223 ± 1.09	−0.606 ± 0.82	0.001	0.002	0.965	0.959	0.004	0.252	0.000	0.000
	U2	−0.203 ± 0.36	0.342 ± 1.96	0.107 ± 0.47	0.074	0.156	0.053	0.043	0.549	0.849	0.002	0.316
	U3	−0.091 ± 0.13	0.329 ± 2.10	−0.102 ± 0.20	0.203	0.484	0.148	0.254	0.293	0.929	0.752	0.653
	U4	−0.413 ± 0.55	0.081 ± 1.03	0.742 ± 1.22	0.000	0.000	0.008	0.000	0.046	0.907	0.000	0.000
	U5	−0.184 ± 0.85	0.067 ± 0.99	0.303 ± 1.22	0.112	0.118	0.264	0.094	0.461	0.384	0.041	0.161

## Data Availability

The data are not publicly available due to privacy or ethical restrictions. The data that support the findings of this study are available from the corresponding authors upon reasonable request and approval by the ethics committee of our hospital.
